# Telomeric i-motifs and C-strands inhibit parallel G-quadruplex extension by telomerase

**DOI:** 10.1093/nar/gkad764

**Published:** 2023-09-23

**Authors:** Roberto El-Khoury, Morgane Roman, Hala Abou Assi, Aaron L Moye, Tracy M Bryan, Masad J Damha

**Affiliations:** Department of Chemistry, McGill University, Montreal, Quebec H3A 0B8, Canada; Department of Chemistry, McGill University, Montreal, Quebec H3A 0B8, Canada; Department of Chemistry, McGill University, Montreal, Quebec H3A 0B8, Canada; Children's Medical Research Institute, Faculty of Medicine and Health, University of Sydney, Westmead, NSW 2145, Australia; Children's Medical Research Institute, Faculty of Medicine and Health, University of Sydney, Westmead, NSW 2145, Australia; Department of Chemistry, McGill University, Montreal, Quebec H3A 0B8, Canada

## Abstract

Telomeric C-rich repeated DNA sequences fold into tetrahelical i-motif structures *in vitro* at acidic pH. While studies have suggested that i-motifs may form in cells, little is known about their potential role in human telomere biology. In this study, we explore the effect of telomeric C-strands and i-motifs on the ability of human telomerase to extend G-rich substrates. To promote i-motif formation at neutral pH, we use telomeric sequences where the cytidines have been substituted with 2′-fluoroarabinocytidine. Using FRET-based studies, we show that the stabilized i-motifs resist hybridization to concomitant parallel G-quadruplexes, implying that both structures could exist simultaneously at telomeric termini. Moreover, through telomerase activity assays, we show that both unstructured telomeric C-strands and telomeric i-motifs can inhibit the activity and processivity of telomerase extension of parallel G-quadruplexes and linear telomeric DNA. The data suggest at least three modes of inhibition by C-strands and i-motifs: direct hybridization to the substrate DNA, hybridization to nascent product DNA resulting in early telomerase dissociation, and interference with the unique mechanism of telomerase unwinding and extension of a G-quadruplex. Overall, this study highlights a potential inhibitory role for the telomeric C-strand in telomere maintenance.

## Graphical Abstract

**Graphical Abstract:**
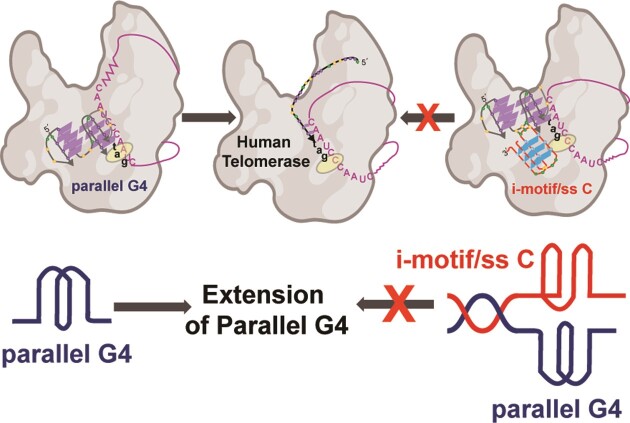

## INTRODUCTION

The human genome includes many cytosine-rich and guanine-rich sequences capable of forming i-motif and G-quadruplex noncanonical structures, respectively. i-Motifs form from intercalated hemiprotonated C:C^+^ base pairs, while G-quadruplexes consist of stacked tetrads of guanine bases. The C-rich and G-rich sequences are prevalent in promoter regions, 5′-UTRs, origins of replication, centromeres, and telomeres (1–6). Evidence from biochemical studies suggests that G-quadruplexes and i-motifs are involved in regulating transcription and replication and maintaining chromosomal telomeres (7–11). Additionally, G-quadruplexes may play a role in modulating splicing and translation (12,13).

Telomeres are the protective ends of chromosomes and span 5–15 kb in length in humans (14). Telomeres shorten with every cellular division, until a critical length that triggers cells to undergo senescence (15,16). In 85–90% of cancers, the enzyme telomerase is overexpressed and is responsible for elongating telomeres and restoring cellular replicative capacity (17). Meanwhile, telomerase expression is repressed in most normal human somatic cells (17).

The human telomeric DNA sequence consists of a tandemly repeated hexameric sequence (GGGTTA) running 5′ to 3′ towards the end of the chromosome, ending in a single stranded overhang that is 12–300 nucleobases in length (18–21). Telomerase is a ribonucleoprotein that can selectively recognize and bind to the overhang through its RNA template and extend it by adding dNTPs using its catalytic reverse transcriptase subunit (hTERT) (22). The G-rich repeat of telomeres is capable of forming G-quadruplexes (23). *In vitro*, the human telomeric sequence has been shown to fold into several G-quadruplex conformations, depending on factors such as the nature of the flanking bases, the nature of the cations in the buffer used, and even the concentration of oligomers present in solution (24–26). For example, the 22-nt human telomeric sequence 5′-AG_3_(TTAGGG)_3_-3′ forms a basket-type mixed antiparallel/parallel conformation in Na^+^ (27), while 5′-G_3_(TTAGGG)_3_T-3′ forms basket-type, two-layer antiparallel G-quadruplexes in K^+^ (28). On the other hand, the human telomeric sequence forms parallel G-quadruplexes in conditions that may mimic those in the nucleus, including high DNA concentration and water depletion (25,29).

While several studies have elucidated the structures that telomeric G-quadruplexes can adopt *in vitro*, the conformation of these quadruplexes *in vivo* remains unclear. Fluorescence microscopy studies using G4-specific antibodies or stabilizing ligands have suggested that G4 structures do form in telomeric regions of fixed human cells (30–33). An antibody specific to parallel G-quadruplexes was shown to localize to telomeres among other regions of chromosomes; this suggests that parallel G-quadruplexes may form in human telomeres (34).

In 1991, it was shown that the telomeric DNA sequence from the ciliate *Oxytricha nova* does not form a substrate for telomerase when it is folded into a G-quadruplex, but this study did not consider the conformation adopted by the G-quadruplexes studied (35). Since then, several ligands have been designed to target and stabilize the telomeric G-quadruplex with the purpose of hindering telomerase activity and inhibiting tumor growth (36–39). However, in 2006, we demonstrated that intermolecular parallel G-quadruplexes ***can*** form substrates of telomerase from the ciliated protozoa *Tetrahymena thermophila* and *Euplotes aediculatus*, while intramolecular antiparallel G-quadruplexes do not (40). More recently, we demonstrated that human telomerase can also recognize and extend intermolecular parallel telomeric G-quadruplexes, as well as intramolecular (monomeric) parallel G-quadruplexes that have been stabilized by 2′-deoxy-2′-fluoro-d-arabinoguanosine (araF-G) substitutions (33,41). In both cases, telomerase first binds and partially unwinds the G-quadruplex, to allow the 3′ end of the DNA to hybridize with the telomerase RNA template, followed by templated nucleotide addition (41). The ability of telomerase to engage with G-quadruplexes is specific to parallel forms; antiparallel or ‘hybrid’ G-quadruplexes are poor substrates for telomerase (33,35,40,42).

**Figure 1.**
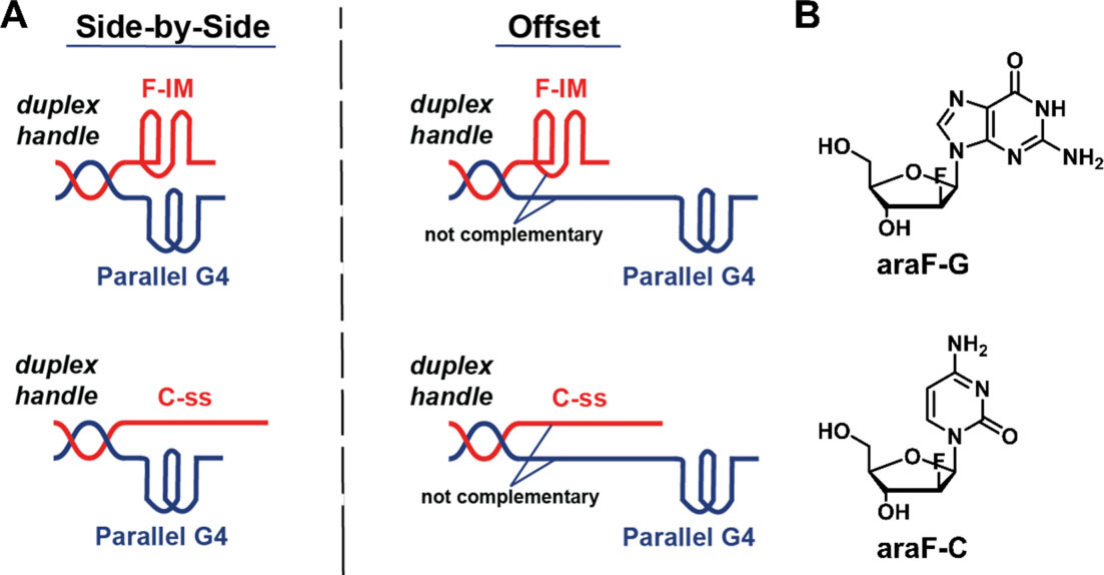
Model systems designed for this study. (**A**) Side-by-Side and Offset models portraying the folding at pH 7 of araF-C rich telomeric sequences into i-motifs (F-IM) and araF-G rich telomeric sequences into parallel G-quadruplexes (G4). At pH 7, the unmodified telomeric C-rich sequence (C-ss) remains single-stranded. (**B**) Structures of araF-G and araF-C nucleoside modifications used to stabilize parallel G-quadruplexes and i-motifs, respectively.

In this study, we explore the extension of monomeric, parallel telomeric G-quadruplexes in the presence of complementary i-motifs, with the two strands tethered by a duplex handle (‘side-by-side’ and ’offset’ models) (Figure 1). Telomeric i-motifs have been shown to form *in vitro* from the C-rich telomeric sequence (5′-TAACCC-3′). However, these are only stable at acidic pH (7,10,43), so their potential effect on the extension activity of telomerase has seldom been explored (44). The study of i-motifs has become increasingly popular, following recent evidence for their formation in cells (45,46). In regard to telomerase, it has been suggested that the formation of telomeric i-motifs can indirectly inhibit the enzyme (44). That study proposed that the formation of the i-motif (triggered by ligand binding, in this case) would free the concomitant complementary strand, allowing it to fold into an antiparallel G-quadruplex which would, in turn, inhibit telomerase. Since human telomerase can extend parallel G-quadruplexes, we have chosen this topology here to study the effect of telomeric i-motifs on telomerase extension activity.

In this work, we use 2′-deoxy-2′-fluoro-d-arabinocytidine (araF-C) modified i-motifs, which are stable at neutral pH (47–49), to directly probe their influence on telomerase extension of parallel G-quadruplex or linear substrates. We have previously demonstrated that araF-C modified i-motifs structurally mimic unmodified DNA-based i-motifs (47). We have also shown that araF-C modified telomeric i-motifs and araF-G modified parallel telomeric G-quadruplexes can simultaneously persist at neutral pH despite their complementarity (48). Here, we use simple model systems in conventional ensemble activity assays to test the influence of i-motif structures and linear C-rich telomeric DNA on telomerase-mediated unwinding and extension of parallel G-quadruplexes. We accompany our activity assays with FRET-based experiments that, unlike our previous NMR studies (48), track the persistence of both G-quadruplex and i-motif structures under telomerase assay buffer conditions and with increasing temperatures. Together, our studies show that both i-motif and unmodified C-rich telomeric DNA strands can block telomerase-mediated addition of multiple telomeric repeats to the concomitant parallel G-quadruplex.

## MATERIALS AND METHODS

### Oligonucleotide synthesis and purification

Oligonucleotides 29-LinG, 33-LinG, HP, pT, pC, pA, C-ss-a and C-ss-b were purchased from Integrated DNA Technologies and the rest were synthesized in our laboratory *via* standard solid-phase synthesis on an ABI 300 DNA synthesizer (Applied Biosystems) (Tables 1–3). The syntheses were done on a 1 μmol scale on Unylinker CPG solid support (Chemgenes) or on dG (N-ibu) 3′-Icaa CPG solid support (Chemgenes) for sequences in Table 3. The monomers used were 5′-O-dimethoxytrityl, 3′-O-[(2-cyanoethyl)-(*N*,*N*-diisopropropyl)]-phosphoramidite derivatives of thymidine, *N*4-acetyl-2′-deoxycytidine (dC), *N*6-benzoyl-2′-deoxyadenosine (dA), *N*2-isobutyryl-2′-deoxyguanosine (dG), *N*2-isobutyryl-2′-deoxy-2′-fluoroarabinoguanosine (araF-G), *N*4-acetyl-2′-deoxy-2′-fluoroarabinocytidine (araF-C) and 5-[*N*-(trifluoroacetylaminohexyl)-3-acrylimido]-2′-deoxyuridine (C5-alkylamino-modified thymidine). The phosphoramidites were used at 0.1 M concentration in acetonitrile. dT, dA, dC, and C5-alkylamino-modified thymidine phosphoramidites were coupled for 200 s, dG for 300 s, araF-C for 600 s and araF-G for 900 s. Cleavage of the oligonucleotide from the CPG and removal of the nucleobase protecting groups were achieved by adding a 1:1 aqueous solution of ammonium hydroxide (30%)/methylamine (40%) at 65°C for 60 min (if cleaving from Unylinker CPG) or for only 15 min (if cleaving from dG 3′-succinate-Icaa CPG). Crude oligonucleotides were purified by anion exchange HPLC on an Agilent 1200 Series instrument using a Protein-Pak DEAE 5PW column (7.5 × 75 mm) at a flow rate of 1 ml/min using a gradient of 0–24% lithium perchlorate buffer (1.0 M) over 30 min at 60°C. Under these conditions, the desired peaks eluted between 22 and 30 min. Samples were desalted on Gel-Pak 2.5 desalting columns according to the manufacturer's protocol. The extinction coefficients of araF-G, araF-C and C5-alkylamino-modified dT were assumed to match those of dG, dC and dT, respectively. Molecular weights were verified by high resolution LC-MS.

**Table 1.** Telomeric G-rich sequences used in this study

Model	Code	Sequence (5′-3′)*
*Parallel G-quadruplexes*		
Side-by-Side	35G3	CACAGATGCGTTTA(fG)GGTTA(fG)(fG)GTTA(fG)(fG)GTTA(fG)GG
Offset	56G3	CACAGATGCGTTTATCTCAGGCGATGGACCGACTT(fG)GGTTA(fG)(fG)GTTA(fG)(fG)GTTA(fG)GG
*Non-parallel (unmodified) G-quadruplexes*		
Offset	56G0	CACAGATGCGTTTATCTCAGGCGATGGACCGACTTGGGTTAGGGTTAGGGTTAGGG
*Linear G-rich telomerase substrates*		
Side-by-Side	29-LinG	Biotin-CACAGATGCGTTTAGGGTTAGGGTTAGGG
Untethered	33-LinG	Biotin-CTAGACCTGTCATCATTAGGGTTAGGGTTAGGG

*(fG): araF-G; Underlined sequences are involved in duplex handle formation.

**Table 2.** Sequences tested for their effect on telomerase extension of parallel G-quadruplexes

Code	Description	Sequence (5′-3′)*
*Tethered (Side-by-Side/Offset)*		
F-IM^#^	IM at pH 7	(fC)(fC)(fC)TAA(fC)(fC)(fC)TAA(fC)(fC)(fC)TAA(fC)(fC)(fC)TTTTCGCATCTGTG
C-ss^#^	ss at pH 7	CCCTAACCCTAACCCTAACCCTTTTCGCATCTGTG
HP	Hairpin	TAGGACTTCGGTCCTATTTTCGCATCTGTG
pT	polyT	TTTTTTTTTTTTTTTTTTTTTTTTTCGCATCTGTG
pA	polyA	AAAAAAAAAAAAAAAAAAAAAATTTCGCATCTGTG
pC	polyC	CCCCCCCCCCCCCCCCCCCCCCTTTCGCATCTGTG
F-IM-a	IM at pH 7	(fC)(fC)(fC)AGT(fC)(fC)(fC)ATA(fC)(fC)(fC)TGT(fC)(fC)(fC)TTTTCGCATCTGTG
C-ss-a	ss at pH 7	CCCAGTCCCATACCCTGTCCCTTTTCGCATCTGTG
F-IM-b	IM at pH 7	(fC)(fC)(fC)(fC)AGT(fC)(fC)(fC)(fC)ATA(fC)(fC)(fC)(fC)TGT(fC) (fC)(fC)(fC)TTTTCGCATCTGTG
C-ss-b	ss at pH 7	CCCCAGTCCCCATACCCCTGTCCCCTTTTCGCATCTGTG
*Untethered*		
F-IM-u^#^	IM at pH 7	(fC)(fC)(fC)TAA(fC)(fC)(fC)TAA(fC)(fC)(fC)TAA(fC)(fC)(fC)T
C-ss-u^#^	ss at pH 7	CCCTAACCCTAACCCTAACCCT

*(fC): araF-C; ^#^F-IM, C-ss, F-IM-u, and C-ss-u correspond to previously studied sequences 35C2, 35C0, 22C2, 22C0, respectively (48). Underlined sequences are involved in duplex handle formation.

**Table 3.** Oligonucleotide sequences used in FRET-based studies

Code	Description	Sequence (5′-3′)*
*Side-by-side*		
F-IM-Cy5	IM at pH 7	(T^Cy5^)(fC)(fC)(fC)TAA(fC)(fC)(fC)TAA(fC)(fC)(fC)TAA(fC)(fC)(fC)TTTTCGCATCTGTG
C-ss-Cy5	ss at pH 7	(T^Cy5^)CCCTAACCCTAACCCTAACCCTTTTCGCATCTGTG
35G3-Cy3	Parallel G-quadruplex	CACAGATGCG(T^Cy3^)TTA(fG)GGTTA(fG)(fG)GTTA(fG)(fG)GTTA(fG)GG

*(fC): araF-C; (fG): araF-G. Underlined sequences are involved in duplex handle formation.

Conjugation of FRET study oligonucleotides (Table 3) to Sulfo-Cy3 or Sulfo-Cy5 dyes was achieved by reacting the (C5-alkylamino)-dT-functionalized oligonucleotides with the NHS ester form of Sulfo-Cy3 or Sulfo-Cy5 dyes (purchased from Lumiprobe). A solution of the oligonucleotide (350 μl, 0.7 mM) in sodium bicarbonate buffer (0.1 M, pH 8.4) was combined with a solution of the Sulfo-Cy dye (NHS ester) in anhydrous DMSO (250 μl, 12.9 mM) and the reaction mixture was left shaking at room temperature for 2.5 h. Samples were evaporated to dryness and the dye-conjugated product was purified by anion exchange HPLC as described earlier. By comparing the area of the HPLC peaks, the yield of the conjugation reaction was 100% (complete conversion) in the case of sulfonated Cy3-conugated G-quadruplex (35G3-Cy3) and 78% in the case of sulfonated Cy5-conjugated C-rich sequences (F-IM-Cy5, C-ss-Cy5). The collected dye-conjugated samples were desalted on Glen-Pak desalting columns according to the manufacturer's protocol, and their molecular weights were confirmed by high-resolution LC-MS.

### Circular dichroism (CD) spectroscopy

Circular dichroism (CD) spectra were recorded on a Chirascan VX spectrometer equipped with a Peltier temperature controller. Samples were prepared at 5 μM concentrations, in 200 μl of KP_i_/KCl buffer (20 mM potassium phosphate, 70 mM potassium chloride) with the desired pH. They were annealed by heating them at 90°C for 5 min, cooling them slowly to room temperature over 3 h, and placing them at 5°C overnight. For samples tested in telomerase assay conditions, telomerase assay buffer was added in an 8:2 ratio of assay buffer to annealed oligonucleotide in KPi/KCl buffer. These samples had a final composition of 51.5 mM Tris, 4 mM KP_i_, 2.5 mM Tricine, 1 mM MgCl_2_, 5 mM dithiothreitol (DTT), 1 mM spermidine, and 164 mM KCl. Five scans were accumulated over the indicated wavelength ranges in a 0.2 cm path length cell. Parameters included a time per point of 0.5 s, sampling of 0.5 s, and bandwidth of 1 nm. Buffers alone were also scanned, and these spectra subtracted from the average scans for each sample. Cuvette path lengths were 1-, 2- or 10-mm. CD spectra were collected in units of millidegrees, and data were smoothed using the Savitzky-Golay function.

Since buffer pH varies with temperature changes, we have dissolved oligonucleotides for thermal denaturation experiments in buffers that have been adjusted for pH at room temperature (22°C), compatible with most telomerase activity assays in this study. Hence, at lower temperatures of spectroscopic acquisition, the pH of KP_i_/KCl buffer would be lower by about 0.08 (50), leading to the stabilization of a marginally smaller population of i-motif. Therefore, our thermal denaturation experiments in KPi/KCl may have captured a smaller population of i-motif. Nevertheless, this does not invalidate our data regarding i-motif stability. As for the Tris-based buffer, its pH would be higher by about 0.9 at the lower temperatures of spectroscopic acquisition (50), which is a significant change. Nevertheless, i-motifs and G-quadruplexes were always pre-folded in KPi/KCl buffer, before adding the Tris-based telomerase buffer at 5 or 10°C. Hence, there would not have been a significant change in the population of i-motifs especially considering the slow folding dynamics of i-motifs.

### UV thermal denaturation experiments

UV-based thermal denaturation experiments were performed on a Cary 100 UV-Vis spectrophotometer equipped with a Peltier temperature controller. Samples were annealed in KP_i_/KCl pH 7 and at 5 μM concentration by heating for 5 min at 90°C, cooling slowly to room temperature over 3 h, and storing overnight at 5°C. For samples tested in telomerase assay conditions, telomerase assay buffer was added in an 8:2 ratio of assay buffer to annealed oligonucleotide in KP_i_/KCl buffer. Acquisitions were performed in 1 cm path-length cuvettes and at 260 nm for hairpin samples, 265 nm for i-motif samples, and 285 nm for G-quadruplex samples. Absorbance values were acquired at 0.5°C/min scanning rate.

### ^1^H NMR spectroscopy

Samples for NMR experiments were dissolved in KP_i_/KCl buffer prepared with 10% D_2_O. Oligonucleotide solutions were prepared at 200 μM and were pre-folded slowly, allowing to cool overnight at 5°C; complementary sequences were only mixed before starting the NMR experiments. NMR spectra were acquired on an 800 MHz Bruker spectrometer and having a cryoprobe with a ^1^H channel. Data were multiplied by a 10.0 Hz line-broadening exponential function and Fourier transformed using TopSpin 4.0.2 software. One dimensional ^1^H NMR spectra were acquired using excitation sculpting for water suppression.

### Fluorescence spectroscopy

Fluorescence spectra were acquired in a Duetta Fluorescence and Absorbance Spectrometer (Horiba Scientific), with an excitation wavelength of 514 nm, an emission range of 530–800 nm, excitation and emission band passes of 5 nm, and an integration time of 0.5 s. Samples were annealed slowly at 5 μM concentrations, in 200 μl of KP_i_/KCl buffer with the desired pH and stored at 5°C overnight. For samples tested in telomerase assay conditions, telomerase assay buffer was added in an 8:2 ratio of assay buffer to annealed oligonucleotide in KP_i_/KCl buffer. Step temperature experiments were set up to record changes in fluorescence spectra with increasing temperatures, with a start temperature of 5°C, and end temperature of 35°C, a step increment of 2.5°C, and an equilibration time of 5 min.

### Direct telomerase activity assays

The following reaction was prepared to give 20 μl per sample: 1 μM of each of the specified G-rich and C-rich oligonucleotides, 50 mM Tris–HCl (pH 6, 7 or 8.5), 1 mM MgCl_2_, 150 mM KCl, 5 mM dithiothreitol, 1 mM spermidine–HCl, 0.5 mM dTTP, 0.5 mM dATP, 5 μM nonradioactive dGTP, 0.17 μM [α-^32^P]dGTP at 20 mCi mL^−1^, 6000 Ci mmol^−1^ (PerkinElmer Life Sciences) and 0.5 μl (∼0.8 nM) of immunopurified human telomerase expressed in HEK293T cells in buffer A (51). Unless otherwise specified, each oligonucleotide strand was heated to 90°C for 10 min then slowly pre-folded in KP_i_/KCl buffer to room temperature and stored overnight at 4°C. Any two strands were only mixed on ice (at a concentration of 10 μM of each strand) just before adding to the assays. Telomerase activity assays were incubated at 20°C for 1 h (or 2 h for experiment in Figure 6). The reaction was quenched by the addition of 80 μl of stop buffer (50 mM Tris–HCl, pH 8.3, 20 mM EDTA and 0.2% SDS) and 300 cpm of a 5′-^32^P-labeled synthetic 30-mer DNA as an internal recovery standard. Products of telomerase extension were recovered with phenol/chloroform extraction followed by ethanol precipitation as described (33). The solution was heated at 90°C for 10 min, and 3 μl was electrophoresed over a 10% polyacrylamide sequencing gel (0.2 mm thick × 40 cm length × 35 cm width, 32-well comb) run in 1× TBE/8 M urea at 75 W. The gel was transferred to filter paper, dried for 30 min at 80°C, exposed to a PhosphorImager screen, visualized on a Typhoon FLA9500 scanner (GE Healthcare Lifesciences) and analyzed using ImageQuant software.

## RESULTS

The G-rich sequences used in this study are listed in Table 1 and either form intramolecular G-quadruplexes or remain as linear G-rich telomerase substrates. Table 2 includes all sequences examined for any effect on telomerase extension of G-rich sequences of Table 1. In particular, the araF-C modified C-rich telomeric sequence is denoted as ‘F-IM’ for its ability to form an i-motif at neutral pH, while the unmodified telomeric sequence is denoted as ‘C-ss’ because it remains single stranded at neutral pH (47,48).

### The araF-N modified G-quadruplexes and i-motifs resist unfolding and hybridization

We have previously shown that ‘side-by-side’ araF-N modified G-quadruplexes and i-motifs (35G3 and F-IM, respectively; Figure 1, left) resist hybridization to form a duplex in 20 mM potassium phosphate buffer (pH 7) containing 70 mM potassium chloride (referred to here as KP_i_/KCl buffer) (48). We used ^1^H NMR spectroscopy because signals corresponding to the imino protons of canonical and noncanonical nucleic acids appear in distinct chemical shift ranges in the spectrum. Specifically, imino proton signals of Watson–Crick–Franklin (WCF) base pairs appear at 12–14 ppm, while those of G-quadruplexes and i-motifs appear at 10–12 ppm and 15–16 ppm, respectively (52). Since the ‘offset’ model (Figure 1, right) has been designed specifically for this study, it is important to compare the behavior of the araF-N modified structures before utilizing the model in biochemical assays with human telomerase.

**Figure 2.**
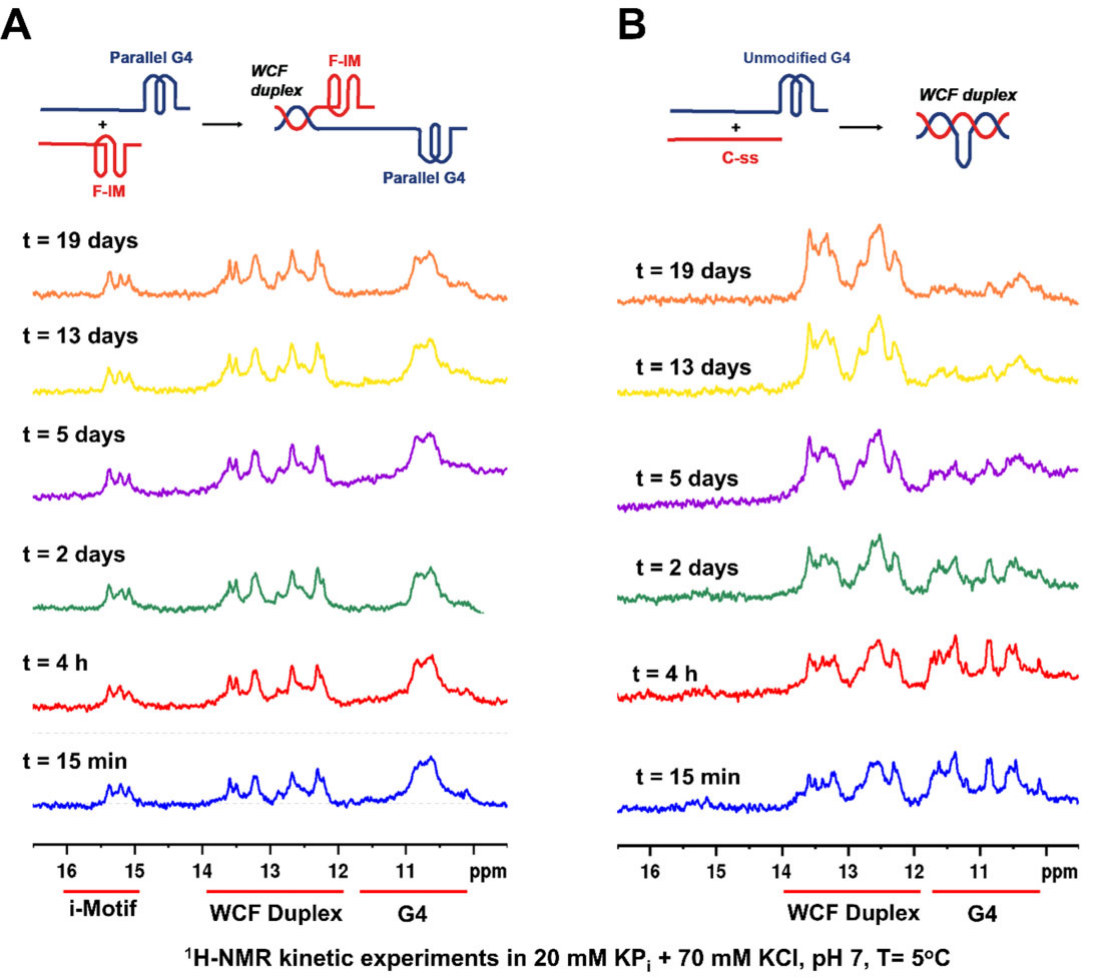
^1^H NMR spectra acquired at different times and at 5°C for an equimolar mixture of ‘offset’ model (**A**) parallel G-quadruplex + F-IM and (**B**) unmodified G-quadruplex + C-ss. Individual strands were pre-folded before mixing in 20 mM KP_i_, 70 mM KCl, pH 7.0.

In kinetic ^1^H NMR experiments, a pre-folded 56-mer parallel G-quadruplex (56G3) was mixed with an equimolar amount of pre-folded araF-C modified F-IM in pH 7 KP_i_/KCl buffer (Figure 2A). As expected, the respective 5′ and 3′ tails of the two sequences hybridized immediately after mixing to yield a duplex handle with characteristic imino protons appearing between 12 and 14 ppm. Moreover, we observed that the signals of the imino protons of the parallel G-quadruplex (10–12 ppm) and the F-IM i-motif (15–16 ppm) persisted with the same intensity for over 19 days at 5°C, thereby resisting any hybridization. This indicates that simultaneous stabilization of the two structures in solution in the ‘offset’ model is akin to that observed previously for the ‘side-by-side’ models (48). As controls, we also mixed the unmodified G-quadruplex (non-parallel) with an equimolar amount of the unmodified C-ss. As expected, we did not see any signal at 15–16 ppm, showing that unmodified C-ss does not fold into an i-motif at pH 7 (Figure 2B). Despite the ‘offset’ nature of the model, we observed a reduction in the intensity of the signals corresponding to the Hoogsteen G:G imino protons (10–12 ppm) with respect to the WCF base pairing region (12–14 ppm), indicating that the population of G-quadruplexes is diminishing as they hybridize to C-ss over time. This result is consistent with the relatively low stability of a non-parallel telomeric G-quadruplex in K^+^ (48). It is likely that the non-complementary middle portion of 56G3 bulges out as C-ss hybridizes to the telomeric portion of 56G3.

### AraF-C modified i-motifs and araF-G modified G-quadruplexes are stable in telomerase activity assay buffer

We have shown that araF-N modified G-quadruplexes and i-motifs can coexist as folded structures in pH 7 KP_i_/KCl buffer at 5°C, but it is important to determine whether they would also remain folded under conditions used in telomerase activity assays, which are done at 20°C in a predominantly Tris-based buffer (see Materials and Methods for total buffer composition).

**Figure 3.**
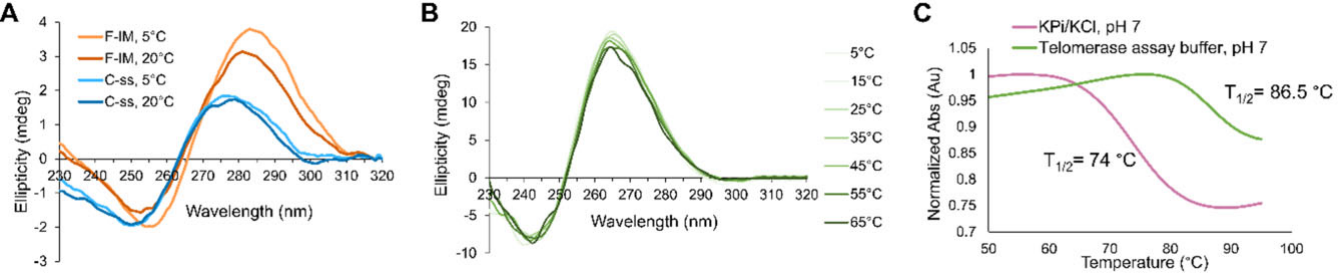
Biophysical studies of G- and C-strands alone. (**A**) CD spectra of F-IM and C-ss in telomerase assay buffer conditions (pH 7), at 5 and 20°C. (**B**) CD spectra of 35G3 G-quadruplex in telomerase assay buffer pH 7 from 5 to 65°C. (**C**) UV-based thermal denaturation data showing changes in normalized absorbance of the 35G3 G-quadruplex with increasing temperature, when measured in KP_i_/KCl buffer pH 7 (pink) or telomerase assay buffer pH 7 (green).

We used CD spectroscopy to analyze the F-IM and C-ss sequences of the ‘side-by-side’ and ‘offset’ models pre-folded in KP_i_/KCl and diluted in telomerase assay buffer. In contrast to its unmodified counterpart, the araF-C modified F-IM displayed a maximum at ∼285 nm and minimum at ∼255 nm at 20°C and pH 7, which are characteristic of i-motif formation (Figure 3A). The slight blue-shift in the positive and negative bands of F-IM at 20°C, compared to 5°C, along with the decrease in their intensity, likely indicate the presence of a small population of single-stranded oligonucleotides or less structured i-motifs. We also analyzed changes in the CD spectra of F-IM and C-ss with increasing temperature (Supplementary Figure S1); only F-IM showed incremental i-motif unfolding with increasing temperature and was completely unfolded at 60°C, in both KP_i_/KCl buffer and telomerase assay buffer, while C-ss was always unfolded.

CD spectra of the araF-G modified G-quadruplex 35G3 (Table 1) indicated that it remains folded in a parallel topology at pH 7, regardless of buffer conditions, with maximum and minimum bands at 265 and 245 nm, respectively (Figure 3B and Supplementary Figure S2).(53) Additionally, UV-based thermal denaturation experiments showed that the G-quadruplex demonstrates high (*T*_1/2_ = 86.5°C) and improved (Δ*T*_1/2_ = +12.5°C) thermal stability in telomerase assay buffer relative to KP_i_/KCl buffer (Figure 3C).

### Studying the simultaneous folding of telomeric i-motifs and parallel G-quadruplexes using FRET

**Figure 4.**
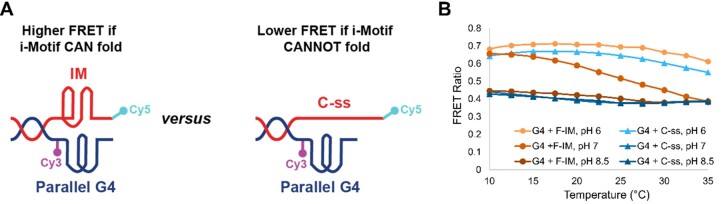
(**A**) Representative ‘side-by-side’ models to measure the extent of telomeric i-motif folding (cy5-conjugated strand) by FRET, in the presence of a concomitant telomeric parallel G-quadruplex (cy3-conjugated strand), 35G3-Cy3. (**B**) FRET ratios calculated from fluorescence emissions across different temperatures following the mixing of pre-folded, equimolar amounts of parallel G-quadruplex, 35G3-Cy3, with either C-ss-Cy5 or F-IM-Cy5 sequences at pH values of 6.0, 7.0, and 8.5. Samples were pre-folded and mixed in KP_i_/KCl buffer, with a duplex concentration of 5 μM, followed by dilution in telomerase assay buffer to make a final duplex concentration of 1 μM.

Studying telomeric i-motif folding in the presence of a concomitant G-quadruplex by CD spectroscopy is complex because of the overlapping peaks corresponding to both structures. Consequently, we designed a FRET-based assay (Figure 4A) to better assess the stability of the i-motif in a representative ‘side-by-side’ model over increasing time and temperature.

In the model, the dyes have been positioned such that the folding of the i-motif would result in a higher FRET signal, while the unfolding of the structure would result in lower FRET. The sequences used in the FRET-based studies are displayed in Table 3. Throughout the fluorescence experiments, the G-quadruplex 35G3-Cy3 is assumed to remain folded in the parallel conformation (Figure 3B, C).

To emulate the conditions that the telomeric i-motif and G-quadruplex would be exposed to when performing telomerase activity assays, we measured fluorescence signals of the FRET-based model after adding telomerase assay buffers to the telomeric strands that have been pre-folded in KPi/KCl and then mixed (Figure 4B). Like CD spectroscopy results (Figure 3 and Supplementary Figure S1), the fluorescence data obtained in this experiment suggest F-IM-Cy5 is mainly folded under assay buffer conditions at 20°C and pH 7.0 (FRET ratio = 0.6). When a similar measurement was done at pH 6.0, both F-IM-Cy5 and C-ss-Cy5 showed i-motif folding, as expected, with FRET ratios around 0.65–0.7. On the other hand, both sequences remained unfolded at basic pH 8.5, with consistent low FRET ratios of ∼0.4 across all temperatures tested. These experiments confirmed that telomeric araF-C i-motifs form and persist under the buffer conditions required to run telomerase activity assays.

To confirm that the duplex handle of the G- and C-rich strands forms upon strand mixing, we monitored changes in fluorescence spectra over time (Supplementary Figure S3) and at the 5°C temperature used for sample preparation in telomerase activity assays. At concentrations relevant to the assays (5 μM), duplex formation occurred almost instantly upon mixing, and the FRET ratio remained stable at 0.62 and 0.4 over several h when F-IM-Cy5 and C-ss-Cy5 sequences were used, respectively. As a control, we also mixed samples that have been diluted 10-fold, and FRET results showed that they required at least 150 min of mixing to ensure complete duplex formation.

### Telomerase extension assays reveal a potential role of telomeric i-motifs and C-strands in regulating telomerase extension of a G-quadruplex substrate

After confirming that the araF-C modified telomeric i-motif (F-IM) remains folded under the pertinent buffer conditions, we performed direct telomerase activity assays to probe any influence the i-motif or linear C-rich telomeric DNA may have on the extension of the parallel G-quadruplex.

**Figure 5.**
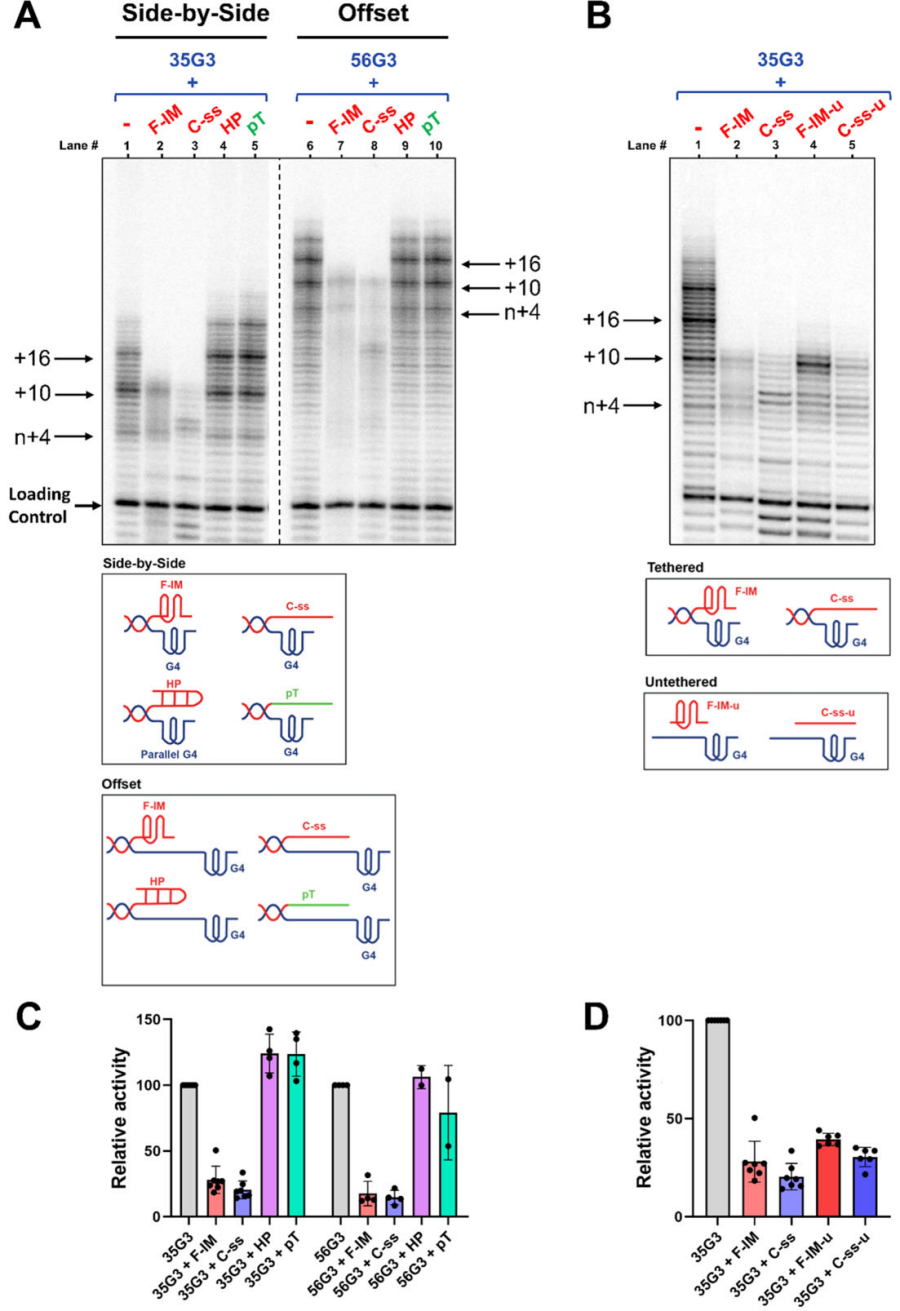
Telomerase extension assays at pH 7 and 20°C, in the presence of radiolabeled α-^32^P-dGTP, analyzed using denaturing polyacrylamide gel electrophoresis. (**A**) The extension products of 1 μM 35G3 or 56G3 alone, or in the presence of 1 μM of F-IM, C-ss, HP or pT. The dashed line represents the omission of irrelevant lanes. (**B**) The extension products of 1 μM 35G3 alone, or in the presence of 1 μM of F-IM, C-ss, F-IM-u or C-ss-u. Loading/recovery control: 5′-^32^P-labeled synthetic 30 nt DNA. (**C**) and (**D**) represent quantitations of relative overall telomerase extension activity in (A) and (B), respectively, in comparison to the extension of 35G3 or 56G3 alone; *n* = 2–7 independent experiments.

The telomerase activity assays of Figure 5 were performed at 20°C and pH 7, which are compatible with F-IM folding (Figures 3 and 4). Lanes 1 and 6 in Figure 5A show the extension pattern of the parallel G-quadruplex of the ‘side-by-side’ and ‘offset’ models, respectively. Both show the typical telomerase extension pattern involving the addition of hexanucleotide repeats, confirming that they are substrates of human telomerase (41). To test the effect of telomeric i-motifs, pre-folded F-IM was mixed with an equimolar amount of pre-folded parallel G-quadruplex of either the ‘side-by-side’ model (lane 2) or the ‘offset’ model (lane 7) before adding telomerase. In both cases, we observed an inhibition of overall telomerase activity, indicated by the decrease in total product intensity, and an abrupt inhibition of telomerase repeat addition processivity, indicated by the failure of telomerase to extend the substrate beyond the first full telomeric repeat (i.e. the addition of 10 nt). Interestingly, C-ss, which cannot fold into an i-motif structure at pH 7, exhibited similar inhibitory effects, also resulting in an inability to add further repeats beyond the first 10–11 nts (Figure 5A, lanes 3 and 8; Figure 5C).

To test whether other tethered nucleic acid secondary structures or single strands affect telomerase activity in the same way as F-IM and C-ss, we synthesized an oligonucleotide with a hairpin (HP) and others with a poly-T (pT), poly-A (pA), or poly-C (pC) single strand at their 5′ ends (Table 2). The hairpin (Supplementary Figure S4) was designed to have a 6-bp stem and a 4-nt loop, such that it would span a similar length as the F-IM 5′ i-motif. Interestingly, none of the sequences had any effect on telomerase activity or processivity (Figure 5A, lanes 4, 5, 9 and 10; Figure 8, lanes 4–7; Figure 5C), suggesting that inhibition of G-quadruplex extension depends on the structure and sequence of the complementary strand.

Next, we tested whether C-rich strands would still affect telomerase extension of parallel G-quadruplexes if they were not held in close proximity to the G-quadruplex through a duplex handle (‘untethered’; Figure 5B). Interestingly, in the presence of 35G3, untethered F-IM-u and C-ss-u (lanes 4 and 5, respectively) inhibited telomerase processivity in the same way as their tethered counterparts (Figure 5B, lanes 2 and 3, respectively; Figure 5D). This suggests that the observed inhibition is not due to simple steric hindrance of the access of telomerase to its substrate.

**Figure 6.**
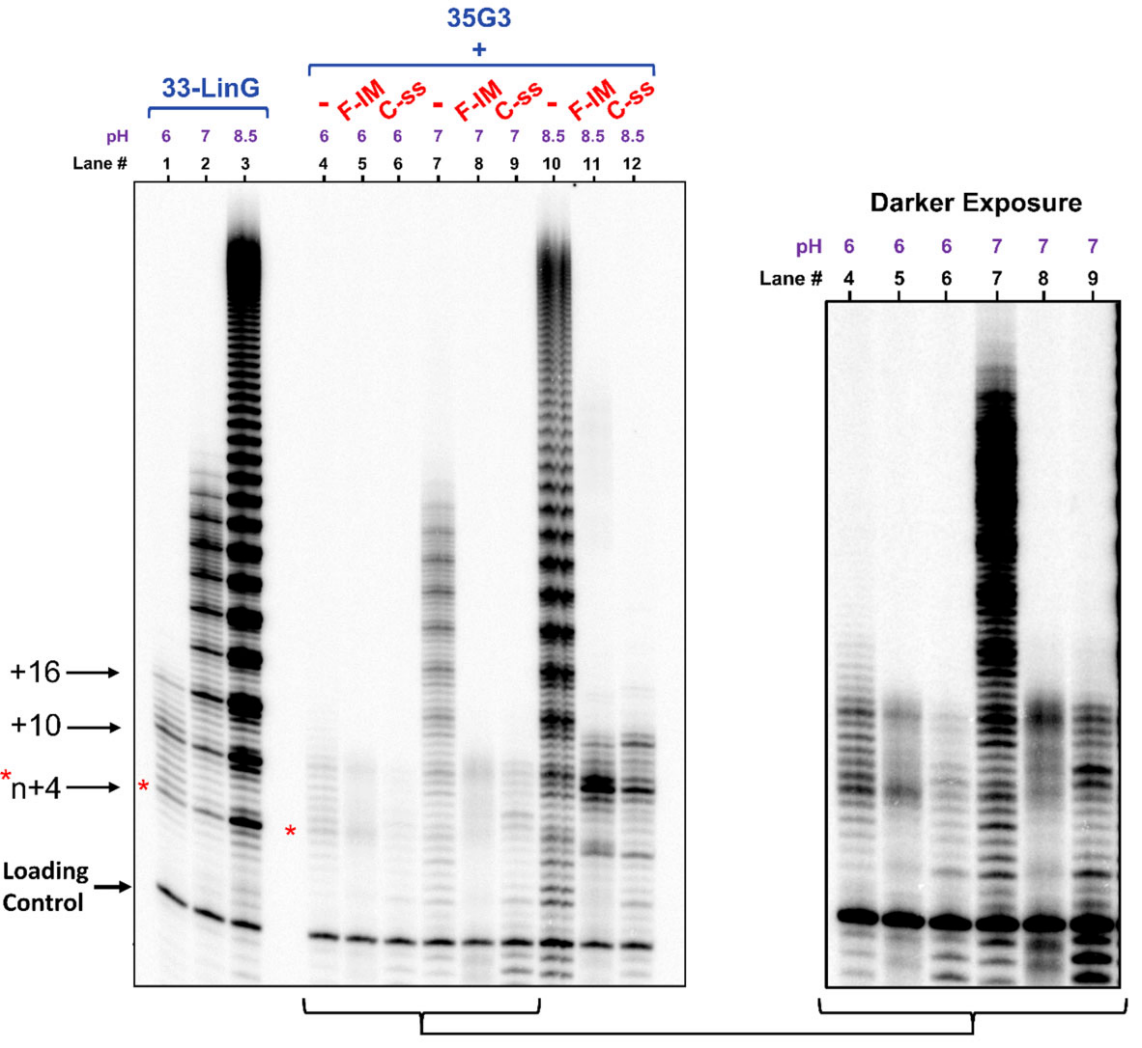
Telomerase extension assays at pH 6, 7, and 8.5 and at 20°C, in the presence of radiolabeled α-^32^P-dGTP. Lanes 1–3: The extension products with varying pH of 1 μM of linear telomeric control (33-LinG) alone, which cannot fold into a G-quadruplex (Table 1). Lanes 4–12: The extension products with varying pH of 1 μM of 35G3 alone, or in the presence of 1 μM of F-IM or C-ss. The inset represents a darker exposure of lanes 4–9. Loading/recovery control: 5′-^32^P-labeled synthetic 30 nt DNA.

Collectively, our results imply that C-strands affect telomerase activity and processivity, regardless of their unstructured (linear) or structured (i-motif) form. Since F-IM is partly unfolded at pH 7, we carried out extension reactions at pH 6, in which F-IM and C-ss are both expected to fold into i-motifs, and at pH 8.5, in which both F-IM and C-ss would be linear (Figure 6). At pH 6, we observed that telomerase has low activity and processivity (lanes 4–6), as confirmed by the poor extension pattern for linear control 33-LinG (lane 1). While the low activity makes it more difficult to detect inhibition, in the presence of both F-IM and C-ss telomerase extension stopped abruptly after 10–11 nt (lanes 5, 6). At a more basic pH of 8.5, we observed that telomerase is more active than at pH 7 (lane 3), yet extension of the G-quadruplex was inhibited in the presence of F-IM or C-ss, which would both be single-stranded at this pH. Overall, the similarity of the results across a pH range of 6–8.5 suggests that the telomeric C-strand inhibits telomerase processivity as either an i-motif or an unstructured single-strand.

### Hybridization may partly explain the inhibition of extension of linear G-strand substrates

**Figure 7.**
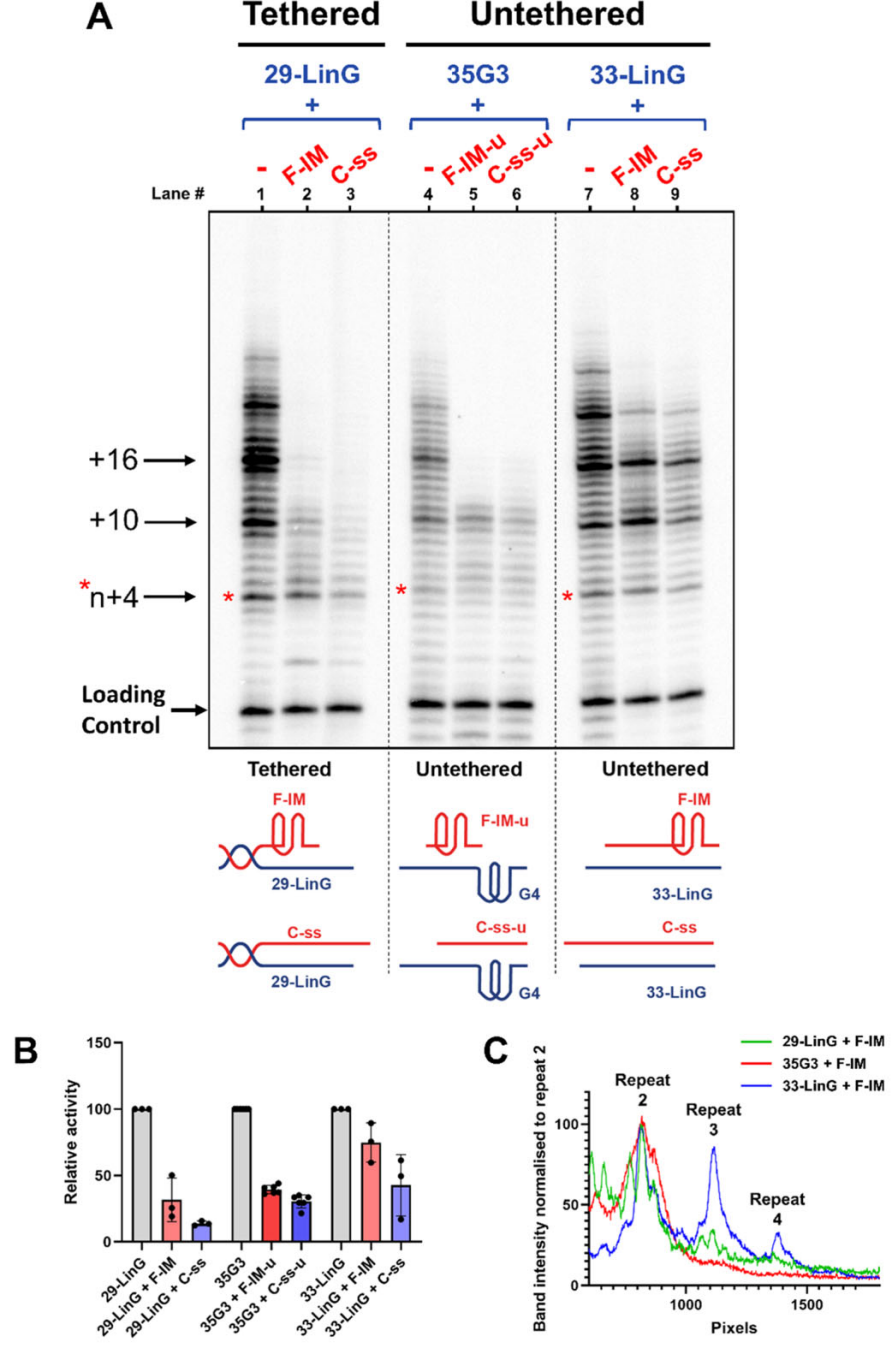
Telomerase extension assays at pH 7 and 20°C, in the presence of radiolabeled α-^32^P-dGTP. (**A**) Bands correspond to extension products of 1 μM ‘side-by-side’ 29-LinG, ‘side-by-side’ parallel G-quadruplex or untethered 33-LinG alone, or in the presence of 1 μM of tethered or untethered F-IM or C-ss. Loading/recovery control: 5′-^32^P-labeled synthetic 30 nt DNA. (**B**) represents quantitation of relative overall telomerase extension activity in (A), in comparison to the extension of 29-LinG, 35G3, or 33-LinG alone. (**C**) represents changes in the intensity of bands corresponding to telomerase extension products, normalized to the intensity of the band corresponding to the addition of the second hexanucleotide repeat.

To explore whether the inhibition observed due to F-IM or C-ss is specific to telomerase extension of G-quadruplexes, we performed telomerase activity assays at pH 7 and 20°C, using a ‘side-by-side’ linear substrate, 29-LinG, (Figure 7A, lanes 1–3) or a linear substrate (33-LinG) which cannot be tethered to the C-strands with a duplex handle (Figure 7A, lanes 7–9), compared to extension of G-quadruplex 35G3 as a control (Figure 7A, lanes 4–6). Neither of the linear substrates can fold into intramolecular G-quadruplexes. Both F-IM and C-ss retained the ability to inhibit telomerase activity and processivity regardless of the nature of the substrate, although inhibition of a linear substrate was more pronounced when it was tethered to the C-strand (Figure 7A, lanes 2, 3, 8 and 9; Figure 7B). Notably, the inhibition of extension of a linear substrate was qualitatively different to the inhibition of extension of a G-quadruplex, since it did not involve an abrupt cessation of processivity after 10 – 12 nt, particularly in the untethered model (33-LinG; lanes 8 and 9). This difference in the mode of inhibition is illustrated in Figure 7C, which shows an example of a lane profile with each of the three substrates in Figure 7A. Both linear substrates show distinct product bands after addition of 3 and 4 repeats (blue, green), whereas the extension of profile of 35G3 (red) is flat after the second repeat (Figure 7C). Moreover, we observed that C-ss inhibited telomerase extension of both linear substrates to a greater extent than F-IM (Figure 7B). Together, these data suggest that the inhibition of extension of linear substrates arises—at least in part—from the hybridization of the complementary C-strand to the linear G-strand, as expected.

### Telomeric i-motif and C-strand mediated inhibition of telomerase activity is sequence-dependent

**Figure 8.**
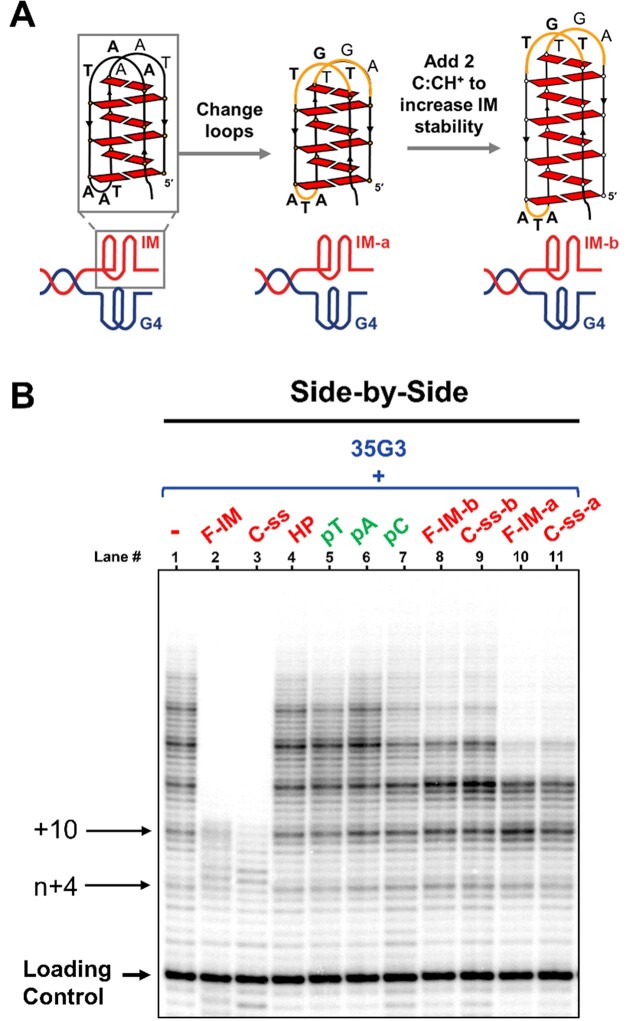
(**A**) Non-telomeric i-motifs, IM-a and IM-b, designed to test the sequence and structure dependence of the inhibitory effects of telomeric i-motifs. (**B**) Telomerase extension assays at pH 7 and 20°C, in the presence of radiolabeled α-^32^P-dGTP. The extension products of 1 μM parallel G-quadruplex (‘side-by-side’) alone, or in the presence of 1 μM of complementary strands (lanes 2–11) were analyzed using denaturing polyacrylamide gel electrophoresis. Loading/recovery control: 5′-^32^P-labeled synthetic 30 nt DNA.

To further understand the determinants of the inhibitory effects of F-IM and C-ss on telomerase extension of a parallel G-quadruplex, we designed two new i-motifs (Figure 8A). In one case, we only modified the loops to generate a non-telomeric i-motif with the same number of C:C^+^ base pairs (6 bp); we denoted the araF-C modified sequence as ‘F-IM-a’ due to its ability to fold into an i-motif at pH 7 and the unmodified sequence as ‘C-ss-a’ because it remained single-stranded at pH 7 (Table 2, Supplementary Figure S5). For the second i-motif, we modified the loops and added two additional C:C^+^ base pairs (8 bp in total) to generate a non-telomeric i-motif with higher thermal stability (Supplementary Figures S5 and S6). These araF-C modified and unmodified sequences are denoted as ‘F-IM-b’ and ‘C-ss-b’, respectively.

Neither F-IM-b nor C-ss-b (Figure 8B, lanes 8 and 9, respectively) had a pronounced effect on telomerase extension of a ‘side-by-side’ G-quadruplex substrate, even though F-IM-b would fold into a more stable i-motif than F-IM (lane 2). On the other hand, results with F-IM-a and C-ss-a (lanes 10 and 11, respectively) show that both sequences inhibited telomerase repeat addition processivity in an identical way. Interestingly, the extension pattern observed in both cases was unique and suggests that telomerase processivity is only inhibited after the third or fourth translocation event, as opposed to the first in the case of F-IM. Therefore, we deduce that the inhibition of parallel G-quadruplex extension due to a concomitant non-telomeric i-motif or C-rich single strand proceeds through a different mechanism than the inhibition caused by F-IM. Results from these assays also demonstrate that the underlying sequence of the i-motif dictates inhibition of telomerase activity.

## DISCUSSION

The findings presented in this study suggest that telomeric i-motifs and linear C-strands can inhibit telomerase extension of parallel G-quadruplex or linear G-rich substrates. Studying the interplay between i-motifs and biological proteins *in vitro* is complicated by the poor stability of i-motif structures at neutral pH (7), and reducing the pH to favour i-motif formation may hinder enzymatic activity, as we have shown with telomerase (Figure 6). By substituting dC with araF-C in the telomeric i-motif sequence, we have stabilized i-motifs enough at neutral pH such that telomerase activity assays can be conducted without the use of exogenous i-motif stabilizing agents, which may introduce bias into the results obtained. While araF-substituted G-quadruplexes and i-motifs may be considered biologically irrelevant, our prior studies have shown that araF substitutions serve as 2′-deoxyribose mimics (49). Specifically, we have shown that araF-modified oligonucleotides are recognized by DNA polymerases (54–56), araF/RNA hybrids can be cleaved by RNase H (57–59), araF duplexes conform to the B-form helical structure (60), araF-modified thrombin-binding aptamers (TBA) bind to thrombin (61), and araF-modified parallel G-quadruplexes are extended by human telomerase (41). Based on these studies and our data herein, we have no reason to believe that araF substitutions introduce bias into our experiments to result in biological irrelevance.

Using FRET-based studies (Figure 4), we have shown that araF-C stabilized i-motifs remain folded in the pH 7 buffer conditions utilized in telomerase activity assays, while tethered to a telomeric G-quadruplex through a duplex handle. While the constructs used do not report on the stability of the parallel G-quadruplex structure, we have demonstrated, using CD spectroscopy and thermal denaturation experiments, that the parallel G-quadruplex has a high thermal stability of 86.5°C in telomerase assay conditions. These results, along with previously obtained findings from our group, suggest that telomerase is capable of directly inducing the unfolding of an araF-G stabilized parallel G-quadruplex (41,48).

**Figure 9.**
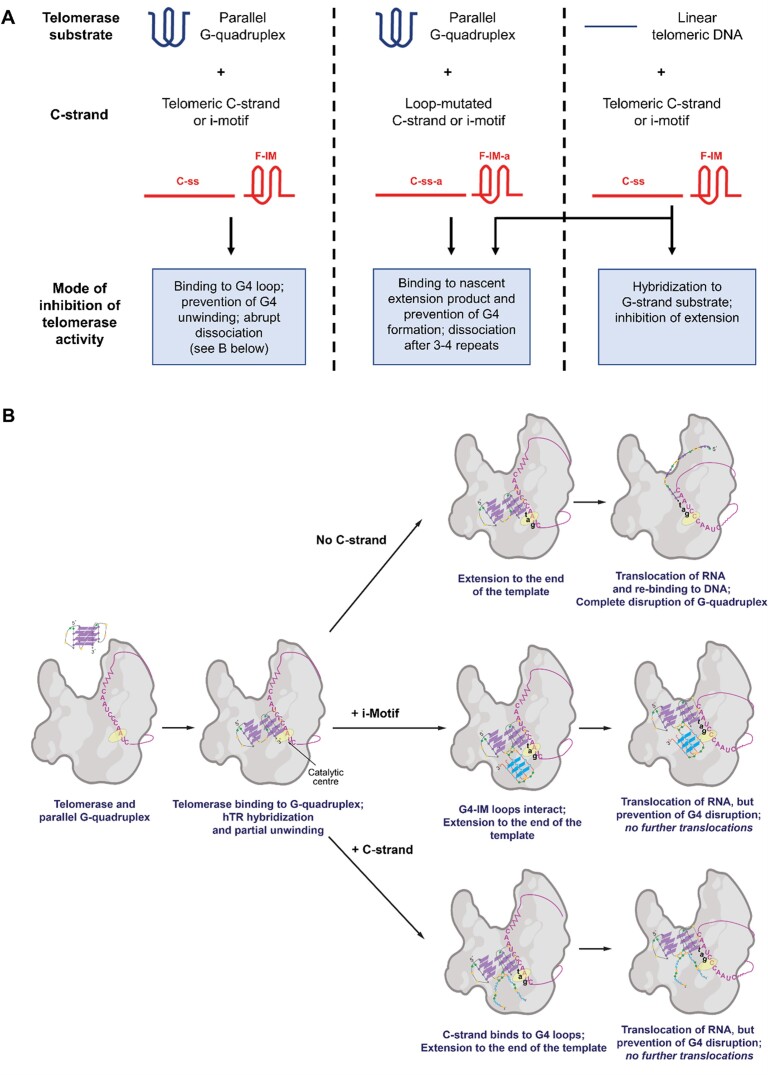
Proposed mechanism for C-strand mediated inhibition of telomerase extension of human telomeric G-rich substrates. (**A**) Summary of proposed modes of inhibition of extension of parallel G-quadruplex or linear G-strand telomerase substrates. (**B**) Proposed models for i-motif and unstructured C-strand mediated inhibition of extension of a G-quadruplex substrate, proceeding via the binding to the loop of a partially unfolded G-quadruplex, leading to abrupt telomerase dissociation.

The telomerase activity assays we have conducted clearly show that both araF-stabilized i-motifs and unmodified C-rich single strands inhibit telomerase activity and/or processivity as it extends a parallel G-quadruplex or linear G-rich substrate. Together, our data suggest that there are at least three modes of telomerase inhibition occurring (Figure 9A). In the case of linear G-strand substrates (29-LinG and 33-LinG), which cannot fold into G-quadruplexes, we hypothesize that the reduction in overall telomerase activity may be in part simply due to hybridization due to C- and G-strand complementarity, resulting in an inability of telomerase to bind its substrate. This mode of inhibition would be expected in the case of a single-stranded telomeric C-strand, but it is possible that the exposed 3-nt loops of a telomeric iM structure could also partially hybridize to the G-strand substrate. In support of this, greater inhibition of extension of a linear substrate was observed in the presence of linear C-strands compared to the i-motif (Figure 7A, B).

Secondly, when using parallel G-quadruplex substrates, we always observe that telomerase efficiently adds the first four nucleotides in a templated manner and undergoes one round of translocation and addition, but its translocation is completely inhibited after the addition of the first 10 nucleotides. Our assays using non-telomeric i-motif sequences (Figure 8) suggest a mechanism for this unusual effect on telomerase processivity. Specifically, non-telomeric sequences that would fold into i-motifs with the same number of C:C^+^ base pairs but in which only the sequences of the loops between C-tracts are changed (F-IM-a and C-ss-a) result in a more gradual inhibition of telomerase processivity, occurring mostly after the addition of 3 or 4 telomeric repeats. This suggests that the sequence of nucleotides between the C-tracts (i.e. those that would constitute the i-motif loops) is essential for the abrupt inhibition of telomerase processivity observed with a G-quadruplex substrate. We have previously demonstrated, using single molecule FRET studies, that telomerase extension of parallel G-quadruplexes proceeds *via* the partial unfolding of the G-quadruplex by telomerase, which frees up its 3′-end to hybridize with the RNA template of telomerase, followed by the catalytic addition of the first 4 nucleotides (41). It is likely that this step of the reaction remains the same in the presence of a complementary C-strand since the single C-strand or i-motif alone cannot unfold araF-G-stabilized parallel G-quadruplexes (Figures 3 and 4) (48). Our FRET studies also demonstrated that the first translocation of telomerase, which would be necessary for the addition of subsequent hexanucleotide repeats, is accompanied by complete unwinding of the G-quadruplex (41). Given that mutation of the C-strand loop sequence was sufficient to relieve the abrupt inhibition of processivity after the addition of the first 10 nucleotides, we hypothesize that telomeric C-strand mediated inhibition proceeds *via* the interaction of i-motif loops, or the corresponding bases of a linear telomeric C-strand, with the remaining loops of the partially-unfolded G-quadruplex, preventing its complete unwinding and hence preventing further template translocations (Figure 9B). This proposed mechanism would explain why this mode of inhibition is unique to a G-quadruplex substrate.

Abrupt inhibition of processivity of extension of the G-quadruplex was observed in the presence of both F-IM and C-ss. We cannot entirely rule out the possibility that the small population of unfolded F-IM at pH 7 and 20°C may be the sole contributor to this mode of telomerase inhibition, especially since the oligonucleotides are present in excess. However, in the presence of either F-IM or C-ss, the G-quadruplex extension products appear identical at pH 6 to those at pH 7 (Figure 6), and both C-strands fold predominantly into i-motifs at pH 6 (Figure 4B), supporting the possibility that the free loops of a telomeric i-motif are sufficient to interfere with telomerase extension of a G-quadruplex.

Thirdly, we hypothesize that the linear C-rich strand may be binding to product DNA as it is formed, leading to the dissociation of telomerase from its product. This hypothesis is supported by a recent study which showed that the folding of nascent DNA product into G-quadruplexes is necessary for proper telomerase processivity (62). It is therefore conceivable that the binding of the linear telomeric C-rich sequence to nascent DNA product would prevent its folding into a G-quadruplex and hence inhibit telomerase processivity. This would be consistent with the observation that the presence of a C-strand results in a decrease in processivity of extension of a linear substrate (Figure 7A). In the case of a G-quadruplex substrate, mutation of the sequence between C-tracts in the C-strand was able to overcome the abrupt processivity block, revealing instead a similar gradual reduction in processivity as seen with linear substrates (Figure 8, lanes 10 and 11). Notably, the same level of processivity inhibition is seen in the presence of either F-IM-a or C-ss-a, and in this case the i-motif loops are not complementary to the G-rich strand, suggesting that much of this mode of inhibition is provided by the linear form of this sequence binding to nascent product DNA. Accordingly, further reduction in complementarity between the 35G3 and non-telomeric C-strand sequences F-IM-b and C-ss-b results in a further reduction in processivity inhibition (Figure 8, lanes 8 and 9).

It is important to note that the models we have used in this study are not designed to simulate the structural arrangement of human telomeres. Among those we have used, the ‘offset’ model (Figure 1), best represents a telomere ending in a G-rich 3′ overhang, and such a model is conceivable since the overhang may be as short as 24 nt (21). While we have found that C-ss and F-IM can inhibit the processivity of telomerase extension of the ‘offset’ parallel G-quadruplex (Figure 5A), implying that the 5′ end of the telomere can interfere with telomerase extension of the 3′ end even from some distance away, the existence of G-quadruplexes and i-motifs at the termini of the telomeres has not yet been directly demonstrated. A G-quadruplex at the end of the 3′ overhang would be mutually exclusive with the ‘t-loop’ arrangement of telomeres, in which the overhang hybridizes with upstream duplex telomeric DNA, although such structures are unwound during DNA replication in S phase (63), which is when telomerase acts. Moreover, recent electron microscopy evidence shows that telomeric chromatin is tightly packed into irregular nucleosomes (64) and may be globular in nature (65); the orientation and structure of the 3′ overhang in the context of this architecture remains to be determined. Nevertheless, the models we have designed serve to isolate two noncanonical telomeric structures shown to fold *in vitro* and investigate their interplay in the presence of human telomerase.

While several lines of evidence point to the role of the G-quadruplexes in regulating cellular processes such as protooncogene transcription and telomere homeostasis, far less is known about the role of i-motifs *in vivo*. Recent studies show that i-motifs can form in cells, and even in the telomeres of chromosomes (45,46). Moreover, like G-quadruplexes, i-motifs have been suggested to regulate protooncogene transcription (66,67), and they are believed to act as on/off switches, in concert with G-quadruplexes, to regulate gene expression (68,69). However, far less is known about the potential role of i-motifs in maintaining human telomeres. Only one study to date has reported that ligand-mediated telomeric i-motif stabilization results in the inhibition of human telomerase activity (44). The authors rely on results from a PCR-based telomerase assay (the TRAP assay) to propose that i-motif formation due to ligand binding frees the complementary G-rich strand, allowing it to fold into a G-quadruplex which, in turn, inhibits telomerase (44). While the results of the study do suggest that telomeric i-motif formation inhibits telomerase activity on the G-strand, the mechanism behind the inhibition was not thoroughly explored and was instead offered speculatively. Moreover, the suitability of TRAP assays to probe for telomerase inhibition is questionable, particularly since ligands have been shown to inhibit the PCR step of the assays (70,71). In contrast, since our work makes use of the parallel G-quadruplex substrate of telomerase, and a direct activity assay in which effects on telomerase processivity are discernible, we could explore alternative mechanisms. Overall, we have found evidence to suggest that the presence of free C-strands at the ends of human telomeres or their folding into i-motifs may interfere with the ability of telomerase to extend either a parallel G-quadruplex or its canonical linear substrate, and the data suggest at least three different mechanisms of inhibition. To our knowledge, there is no *a priori* reason for telomeric C-strands to be single-stranded in cellular conditions: they would be hybridized to the complementary G-strand during and after replication, and the resulting dsDNA/G-overhang junction is recognized by both Apollo and Exonuclease I enzymes involved in C-strand resection (18,72–74). Indeed, our work suggests that if conditions favor relaxation of the duplex, unhybridized C-strands would inhibit telomerase activity, thereby adversely affecting cells that express the enzyme. Given the correlation between telomerase overexpression and cancer cell immortality, our work warrants additional studies to investigate the potential utility of telomeric i-motifs or linear C-strands as tools for telomerase inhibition.

## Supplementary Material

gkad764_Supplemental_File

## Data Availability

The data underlying this article are available in the article and in its online supplementary material.

## Notes

Present address: Roberto El-Khoury, Eli Lilly and Company, 450 Kendall St, Cambridge, MA02142, USA.

Present address: Hala Abou Assi, Base4 Biotechnology, 4324 S Alston Ave, Durham, NC 27713, USA.

Present address: Aaron L. Moye, Stem Cell Program and Divisions of Hematology/Oncology and Pulmonary & Respiratory Diseases, Boston Children's Hospital, Harvard Stem Cell Institute, and Department of Genetics, Harvard Medical School, Boston, USA.
