# Supplementary material for: Telomeric i-motifs and C-strands inhibit parallel G-quadruplex extension by telomerase
Source: Nucleic Acids Res. 2023 Sep 23;51(19):10395–410. doi: 10.1093/nar/gkad764 (PMC10602923; doi:10.1093/nar/gkad764)
Supplement: gkad764_Supplemental_File [file gkad764_supplemental_file.docx]

**Supplementary Data**

**Telomeric i-motifs and C-strands inhibit parallel G-quadruplex extension by telomerase**

**AUTHORS**

Roberto El-Khoury^1^, Morgane Roman^1^, Hala Abou Assi^1,#^, Aaron L. Moye^2,†^, Tracy M. Bryan^2,^*, and Masad J. Damha^1,^*

^1^ Department of Chemistry, McGill University, Montreal, Quebec, H3A 0B8, Canada

^2^ Children’s Medical Research Institute, Faculty of Medicine and Health, University of Sydney, Westmead, NSW 2145, Australia

* To whom correspondence should be addressed. Tel: +1 514 396 6940
Email: masad.damha@mcgill.ca

Correspondence may also be addressed to. Tel: +61 2 8865 2800
Email: TBryan@cmri.org.au

^#^Present address: Base 4 Biotechnology, 4324 S Alston Ave, Durham, NC 27713, United States

†Present address: Stem Cell Program and Divisions of Hematology/Oncology and Pulmonary & Respiratory Diseases, Boston Children’s Hospital, Harvard Stem Cell Institute, and Department of Genetics, Harvard Medical School, Boston, United States


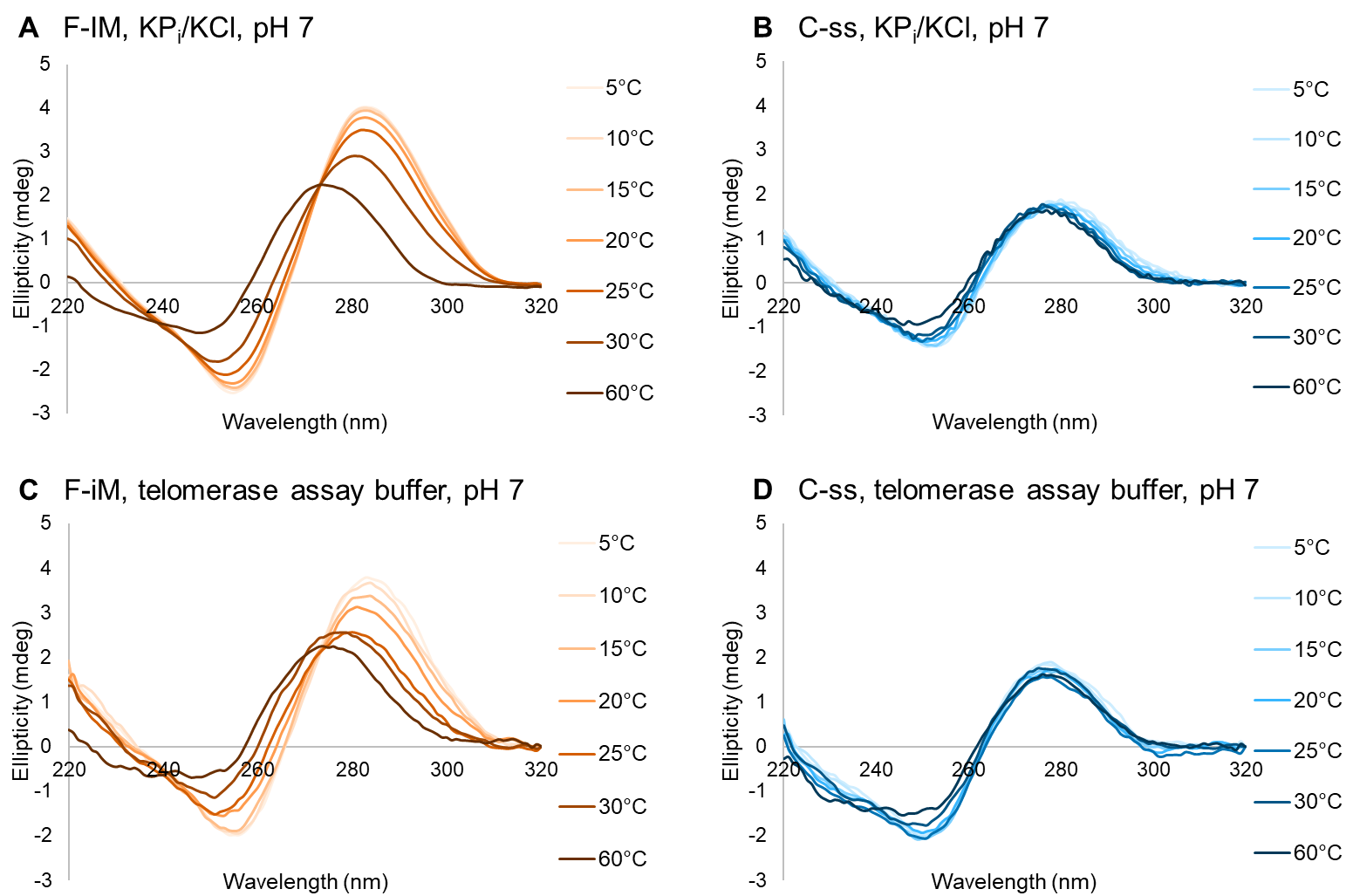


**Figure S1.** Changes in CD spectra of F-IM and C-ss sequences (5 μM) with increasing temperatures in both KP_i_/KCl pH 7 (A&B) and telomerase assay buffer pH 7 (C&D). KP_i_/KCl refers to 20 mM potassium phosphate, 70 mM potassium chloride, while telomerase assay buffer contains 51.5 mM Tris, 4 mM KP_i_, 2.5 mM Tricine, 1 mM MgCl_2_, 5 mM dithiothreitol (DTT), 1 mM spermidine, and 164 mM KCl.


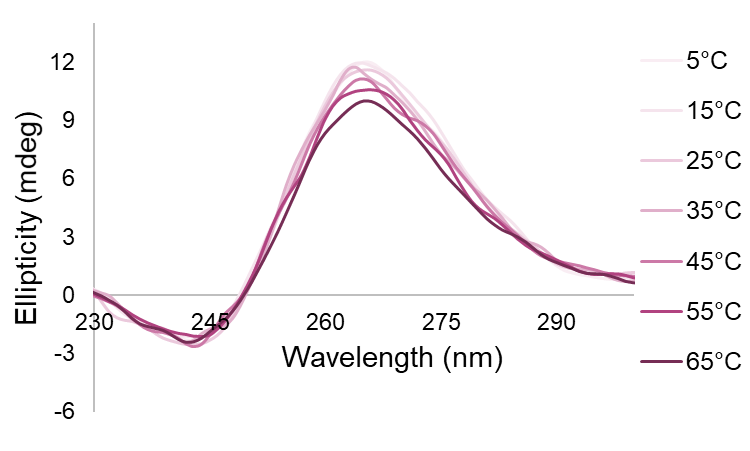


**Figure S2.** CD spectra of 35G3 G-quadruplex (5 μM) in 20 mM KP_i_, 70 mM KCl pH 7 from 5 to 65°C.


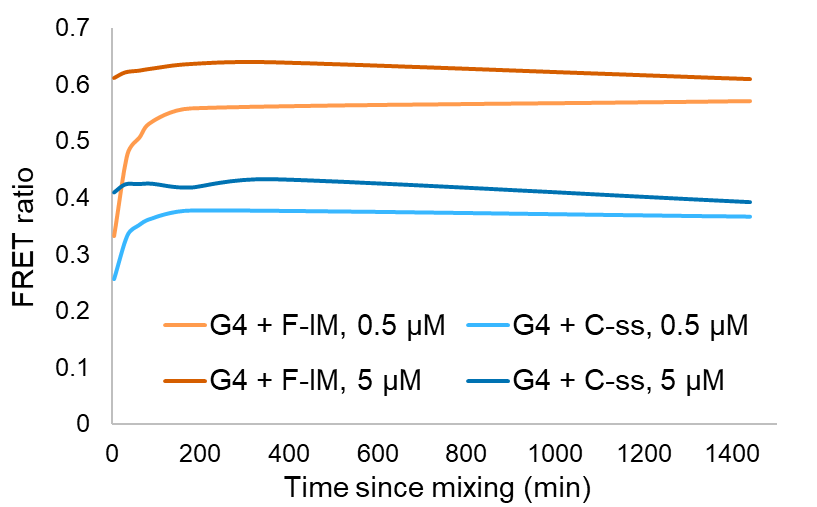


**Figure S3.** FRET ratios calculated from fluorescence emissions at 5°C, over 1440 minutes, following the mixing of the parallel G-quadruplex sequence 35G3 with either F-IM or C-ss sequences. Experiments were conducted in 20 mM KP_i_, 70 mM KCl buffer pH 7.0, with a final duplex concentration of either 0.5 or 5 μM.


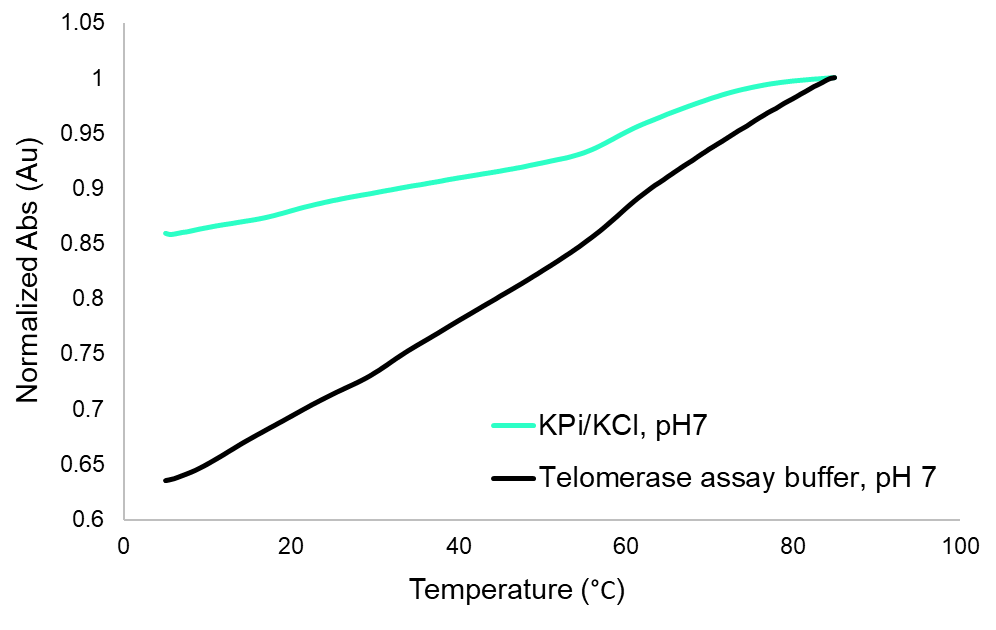


**Figure S4.** UV-based thermal denaturation data showing changes in normalized absorbance of the HP (5 μM) with increasing temperature, when measured in KP_i_/KCl buffer pH 7 (blue-green) or telomerase assay buffer pH 7 (black). KP_i_/KCl refers to 20 mM potassium phosphate, 70 mM potassium chloride, while telomerase assay buffer contains 51.5 mM Tris, 4 mM KP_i_, 2.5 mM Tricine, 1 mM MgCl_2_, 5 mM dithiothreitol (DTT), 1 mM spermidine, and 164 mM KCl.


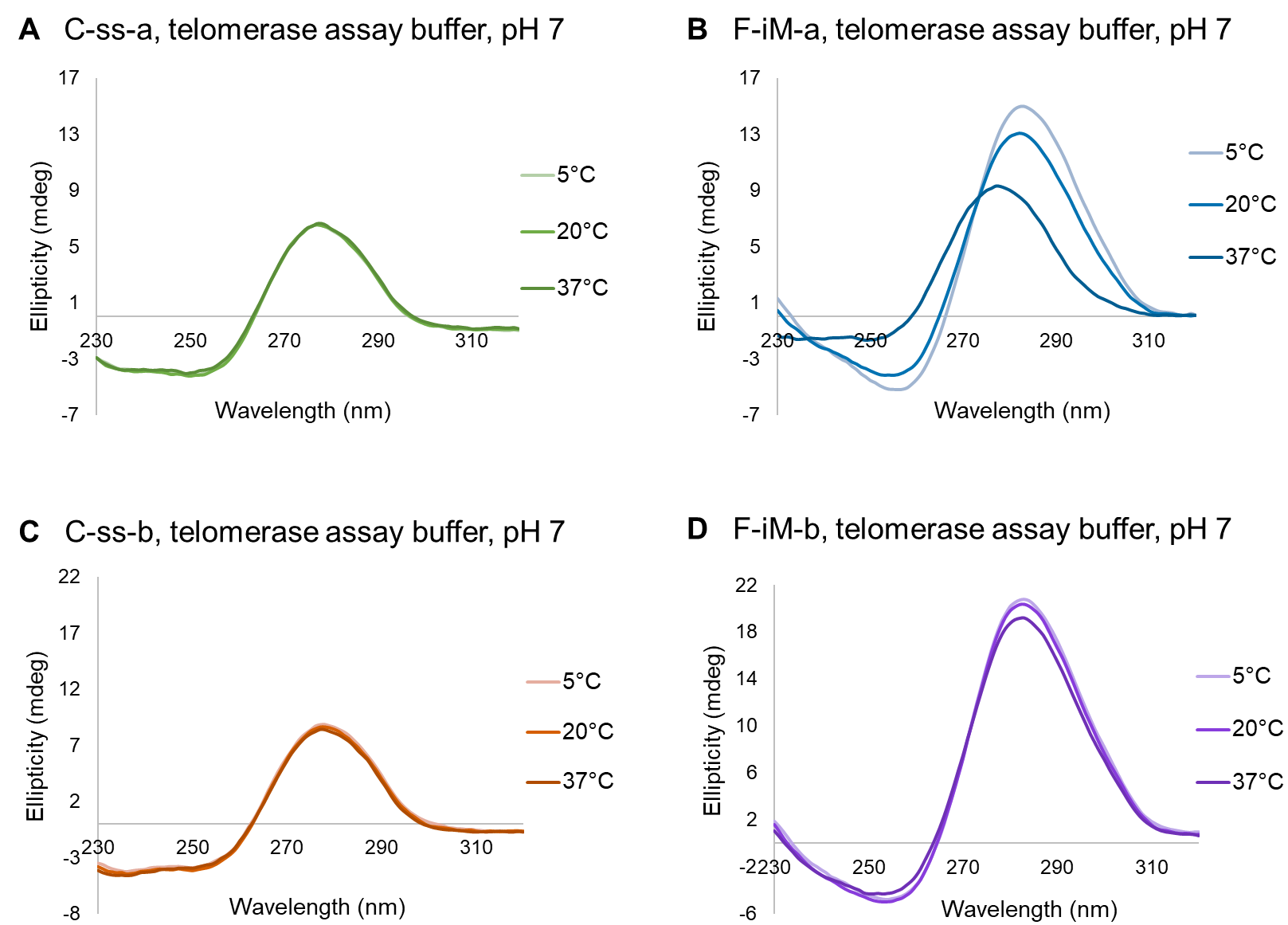


**Figure S5.** CD spectra in telomerase assay buffer (pH 7) of 5 μM concentrations of (A) C-ss-a, (B) F-IM-a, (C) C-ss-b, and (D) F-IM-b at three different temperatures. Telomerase assay buffer contains 51.5 mM Tris, 4 mM KP_i_, 2.5 mM Tricine, 1 mM MgCl_2_, 5 mM dithiothreitol (DTT), 1 mM spermidine, and 164 mM KCl.


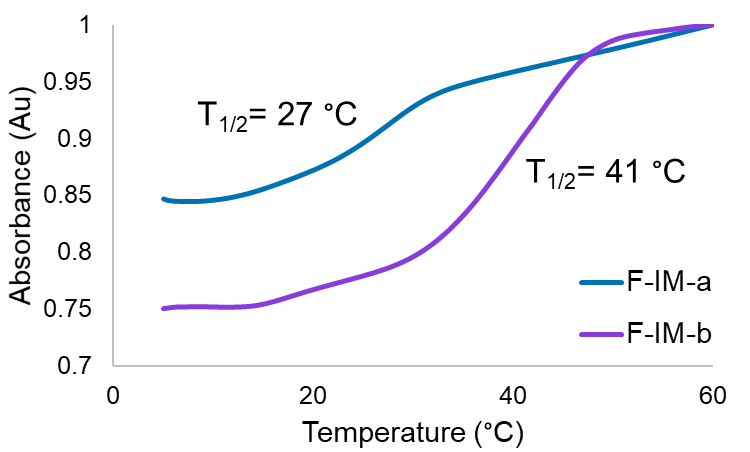


**Figure S6.** UV-melting analysis of 5 μM F-IM-a and F-IM-b in telomerase assay buffer (pH 7). Telomerase assay buffer contains 51.5 mM Tris, 4 mM KP_i_, 2.5 mM Tricine, 1 mM MgCl_2_, 5 mM dithiothreitol (DTT), 1 mM spermidine, and 164 mM KCl.
